# Functional activation of the AKT-mTOR signalling axis in a real-world metastatic breast cancer cohort

**DOI:** 10.1038/s41416-024-02852-y

**Published:** 2024-09-25

**Authors:** Deepika Prasad, Elisa Baldelli, Edik M. Blais, Justin Davis, Emna El Gazzah, Claudius Mueller, Alison Gomeiz, Aisha Ibrahim, Avani Vinayak Newrekar, Brian A. Corgiat, Rick Dunetz, Emanuel F. Petricoin III, Qi Wei, Mariaelena Pierobon

**Affiliations:** 1 School of Systems Biology, George Mason University, Manassas, VA USA; 2 Center for Applied Proteomics and Molecular Medicine, George Mason University, Manassas, VA USA; 3 Perthera Inc, McLean, VA USA; 4 Theralink Technologies Inc, Golden, CO USA; 5 Side-Out Foundation, Fairfax, VA USA; 6 Department of Bioengineering, George Mason University, Fairfax, VA USA

**Keywords:** Molecular medicine, Breast cancer

## Abstract

**Background:**

Mutations of the *PIK3CA/AKT/mTOR* axis are common events in metastatic breast cancers (MBCs). This study was designed to evaluate the extent to which genetic alterations of the *PIK3CA/AKT/mTOR* can predict protein activation of this signalling axis in MBCs.

**Methods:**

Molecular profiles were generated by CLIA-certified laboratories from a real-world evidence cohort of 171 MBC patients. Genetic alterations of the *PIK3CA* pathway were measured using next-generation sequencing. Activation levels of AKT and downstream signalling molecules were quantified using two orthogonal proteomic methods. Protein activity was correlated with underlying genomic profiles and response to CDK4/6 inhibition in combination with endocrine treatment (ET).

**Results:**

Oncogenic alterations of the *PIK3CA*/*AKT*/*PTEN* pathway were identified in 49.7% of cases. Genomic profiles emerged as poor predictors of protein activity (AUC:0.69), and AKT phosphorylation levels mimicked those of mutant lesions in 76.9% of wild-type tumours. High phosphorylation levels of the PI3K/AKT/mTOR downstream target p70S6 Kinase (T389) were associated with shorter PFS in patients treated with CDK4/6 inhibitors in combination with ET (HR:4.18 95%CI:1.19–14.63); this association was not seen when patients were classified by mutational status.

**Conclusions:**

Phosphoprotein-based measurements of drug targets and downstream substrates should be captured along with genomic information to identify MBCs driven by the PI3K/AKT/mTOR signalling.

## Introduction

Breast cancer remains the second-leading cause of cancer-related death for women in the United States and mortality is almost exclusively associated with the development of metastatic disease. Approximately 70% of advanced breast cancers are characterised by the expression of the oestrogen and/or progesterone hormone receptors (HR) and lack of overexpression of the human epidermal growth factor receptor 2 (HER2) [1]. The introduction of endocrine therapy (ET) in combination with a cyclin-dependent kinase 4 and 6 (CDK4/6) inhibitor has significantly impacted survival for breast cancer patients [2–4]. This therapeutic regimen has become the standard of care first-line treatment for newly diagnosed ER+/HER2- metastatic breast cancers (MBC). However, while this treatment has significantly improved progression-free and overall survival in MBC, most patients develop resistance.

Aberrant activation of the phosphatidylinositol 3-kinase (PI3K)/AKT/mTOR signalling pathway has been identified as a key mechanism of resistance to treatment in cancer, including ET with or without a CDK4/6 inhibitor in MBC patients [5–9]. Activating mutations of *PIK3CA* have been detected in ~40% of HR+/HER2- breast cancers, and more than 50% of breast cancer patients harbour oncogenic alterations of at least one member of the *PIK3CA* pathway [10, 11]. As a result, selective inhibitors aiming at modulating the activity of signalling proteins belonging to the PI3K/AKT/mTOR axis have received regulatory approval as treatment options in the MBC setting.

The mTOR complex-1 inhibitor everolimus, a targeted agent that specifically interferes with the activation of a critical downstream node of the PI3K/AKT signalling axis, was approved in 2012 in combination with exemestane for its superior efficacy in MBC patients with progressive disease compared to ET alone regardless of patients’ *PIK3CA* mutations status [12–14]. The alpha-selective PI3K inhibitor alpelisib was also recently approved as a therapeutic option in combination with fulvestrant in MBC patients whose tumours harbour oncogenic alterations of the *PIK3CA* gene, including patients that have progressed after receiving ET in combination with a CDK4/6 inhibitor [15, 16]. However, preclinical work by Palafox et al. has suggested that PI3K inhibition in tumours with acquired resistance to ribociclib can be independent of the *PIK3CA* mutation status of the tumour [5].

Based on the outcome of the randomised phase 3 CAPItello-291 trial, the Food and Drug Administration has recently approved capivasertib in combination with fulvestrant for MBC patients who develop disease progression to CDK4/6 inhibition in combination with ET. The approval is restricted to patients whose tumours harbour oncogenic alterations of the *PIK3CA*, *AKT*, or *PTEN* gene. However, an exploratory analysis of the trial suggests that capivasertib may also be effective in *PIK3CA*/*AKT*/*PTEN* non-altered tumours [17]. Similarly, a secondary biomarker analysis of the FAIRLANE trial assessing the efficacy of ipatasertib in combination with paclitaxel in the neoadjuvant setting in triple-negative breast cancers (TNBC) has suggested that phosphorylation levels of AKT were associated with clinical benefit even in the absence of genetic alterations of *PIK3CA*, *AKT*, or *PTEN* [18]. Likewise, several studies assessing the role of the AKT/mTOR axis in promoting resistance to ET in combination with a CDK4/6 inhibitor have suggested an essential role for genomic-independent activation of this signalling pathway in response to treatment [5, 6, 19]. Thus, a pressing question remains unanswered in the clinic: should agents targeting signalling molecules of the AKT/mTOR axis, like alpelisib and capivasertib, be solely administered to patients whose tumours harbour genomic alterations of genes encoding for members of this pathway? To answer this question, we have conducted an observational retrospective study using a real-world evidence (RWE) cohort of MBC patients and have correlated activation levels of signalling molecules of the AKT/mTOR axis with underlying genomic alterations of members of the *PIK3CA* pathway using a proteogenomic approach. To increase the translational potential of our work, genomic and phospho-proteomic profiles were generated by commercial and accredited laboratories.

## Methods

### Patients’ enrolment and samples’ collection

A retrospective cohort of 176 MBC patients was analysed from Perthera’s RWE database, a platform where patients across the United States are registered on an IRB-approved observational protocol (WCG IRB Protocol ID: PCT-01-012) and encouraged to undergo multi-omic profiling including a comprehensive next-generation sequencing (NGS) testing panel using a tumour sample at a Clinical Laboratory Improvement Amendments certified (CLIA)/College of American Pathologists (CAP)-accredited commercial laboratory. Patients entered the study through physician referrals directly to Perthera (McLean, VA) or via the Side-Out Foundation MCB programme, a patient-centred initiative sponsored by the Side-Out Foundation and contracted to Perthera. Clinical features and treatment outcomes were manually curated from physician’s notes, pathology and radiology reports and other medical records obtained via periodic records requests to patients’ treating institutions [20–22]. HR and HER2 status were assessed as a part of the routine care by the treating physicians and collected along with other clinical-pathological information. Patients entered the study voluntarily and each participant provided informed consent. The study was conducted following the Declaration of Helsinki guidelines. Patients with a confirmed diagnosis of MBC aged 18 years or older were eligible to enter the study.

Molecular testing was performed on Formalin-Fixed Paraffin-Embedded biospecimens collected at time of study enrolment or on archived tissue samples from surgical resections, core needle biopsies and fine needle aspirates. Generally, biopsies acquired within a year of testing were utilised for molecular profiling. Tissue sections were prepared, randomised and sent to CAP/CLIA-accredited laboratories for genomic and proteomic testing.

### Next-generation sequencing

Commercial laboratories performed genomic testing for the presence and significance of pathogenic variants. The majority of NGS results were reported by Foundation Medicine (*n* = 78). Other comprehensive NGS panels with at least 150 genes were also generated by Caris Life Science (*n* = 24), Personal Genome Diagnostics (PGDx) (*n* = 7), Tempus (*n* = 11), other platforms (*n* = 12) and unknown (*n* = 31) (Fig. S1 for more information on overlapping panels). NGS-based results were harmonised into a structured format and reviewed within Perthera’s Virtual Molecular Tumour Board alongside patients’ medical and treatment histories [20].

Mutation frequency analyses were performed at the individual gene- and PI3K/AKT/mTOR pathway level. Genes included in the pathway-level analysis were identified using the Pathcards database [23]. To assess whether mutation rates in the Side-Out cohort matched those of previously analysed datasets, our findings were compared against two publicly available MBC cohorts retrieved from the Genomics Evidence Neoplasia Information Exchange (GENIE) [24–28]. The Inserm cohort included whole exome sequencing data collected from 216 MBC patients [27]. The Memorial Sloan Kettering Cancer Center (MSK) cohort included targeted exome sequencing data collected from 1116 individual HR+/HER2- MBC patients; data were consolidated as a single entry for patients with multiple biopsies (*n* = 195) [28]. Pathogenic alterations in the GENIE cohorts were determined using the Ensembl calculated variant consequences guide [29].

### Immunohistochemistry

Quantification of phosphorylated AKT (pAKT) was performed on 32 FPPE samples using immunohistochemistry (IHC). Levels of pAKT were measured by Neogenomics using the Leica antibody clone LP18. Samples were scored on a dichotomous scale (high/low) and samples with staining intensities of 1+ in at least 50% of tumour cells were classified as pAKT high/positive.

### Reverse phase protein microarray

Activation levels of six signalling molecules belonging to the AKT/mTOR signalling pathway were quantified in 69 patients using the Reverse Phase Protein Microarray (RPPA) assay performed by the CLIA-certified commercial laboratory Theralink Technologies. Pure tumour epithelia were isolated from the surrounding cells using laser capture microdissection, and arrays were constructed and processed as previously described [30, 31]. Primary antibodies were used to recognise the following phosphoproteins: AKT (S473) and (T308), mTOR (S2448), 4EBP1 (S65), p70S6 Kinase (T389) and S6 Ribosomal Protein (S6RP) (S235/236) (Cell Signalling, CST4060, CST4056, CST5536, CST13443, CST9234 and CST4856). Samples were analysed as previously described [32]. Normalised intensity values of each sample were fit to an analyte-specific reference standard curve. The resulting values were compared to a breast cancer reference population to determine each patient’s sample percentile score.

### Breast cancer cell line subculture and treatment

Two commercially available HR+/HER2- breast cell lines, namely T47D and MCF-7, were obtained from the American Type Culture Collection (ATCC) and cultured in medium (RPMI-1640 and Eagle’s MEM, respectively) supplemented with 10% foetal bovine serum (ATCC) and human recombinant insulin in zinc solution (Thermo Fisher Scientific), per manufacturer’s instructions. Abemaciclib-resistant T47D models were generated by exposing cells to increasing amounts of the inhibitor. The IC50 concentration of abemaciclib (Selleckchem) (0.2 μM; data not shown) was used as a starting point and the amount of drug was doubled every 2 weeks to a final concentration of 2 μM, as previously described [33–35]. Cells were plated in a 96-well plate (Corning) and treated in technical replicates (*n* = 4) when they reached 80% confluency with serial dilutions of the pan-AKT inhibitor capivasertib (range 0.01–10 μM) and the PI3K inhibitor buparlisib (range 0.007–1 μM) (Selleckchem). Drug testing was performed with cells at passages 7 or 8. Cell viability was assessed 72 h after treatment using the commercially available CellTiter-Glo assay (Promega) [36]. IC50 values were calculated using a four-parameter curve fit method using GraphPad v9.5.1. For each data point, DMSO-normalised cell viability data were displayed along with standard errors of the mean.

## Statistical analysis

Python’s Pandas and R v4.1.3 packages were used to perform data parsing and analyses of clinical, pathological and molecular data. Packed bubble graphs generated in Tableau v2.1 were used to display gene frequencies. The chi-square test of independence and Fisher’s exact tests were performed in the native stats R package v4.1.3 to understand relationships between categorical variables. The most appropriate test was selected based on the number of counts in each comparison group. Frequencies were displayed using mosaic plots generated with ggplot, ggmosaic, tidyverse, ggpubr and devEMF packages in R. Matrices of genomic alterations were generated in Python and data were visualised in Matlab vR2023b. Unsupervised hierarchical clustering of the RPPA continuous data was performed in Jmp Pro v17.1.0 using Ward’s method. Receiver operating characteristic (ROC) curves and area under the curve (AUC) were calculated using SPSS v28. Non-parametric Mann-Whitney and Kruskal-Wallis tests were performed in Prism v10.0.0, and continuous data were displayed as violin plots. Python Packages like Pandas, Seaborn, Matplotlib were used for data visualisation. Survival analysis and Kaplan-Meier curves were generated in R using the survival v3.5.5 and survminer v0.4.9 packages. The alpha level of significance for all comparisons was set at 0.05.

## Results

### Cohort description and patients’ clinical-pathological characteristics

**Fig. 1:**
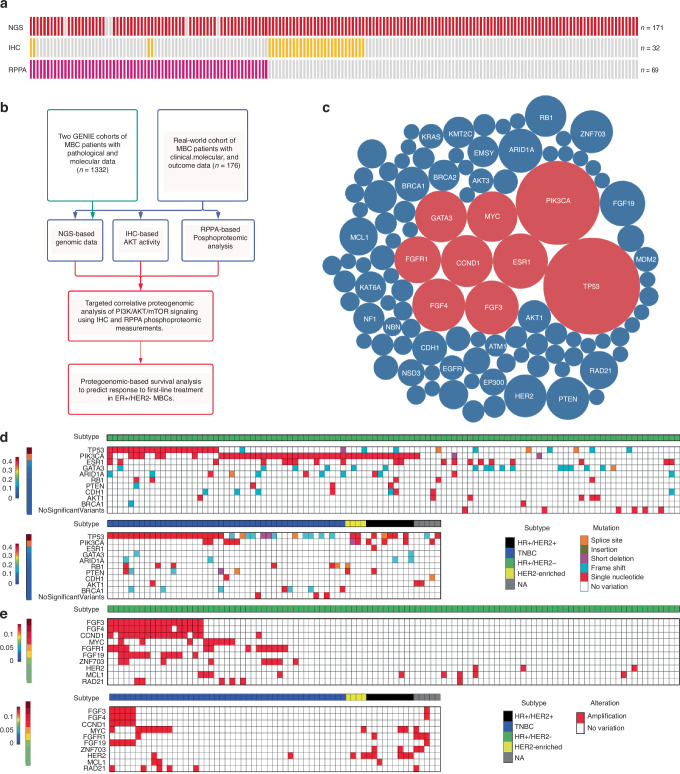
Study workflow and genomic characterisation of 176 MBCs retrospectively identified from a real-world evidence database. Summary of the molecular information collected from each of the 176 patients enroled in the study (**a**). Overall study design along with the molecular information available for each cohort and data processing pipeline (**b**). Packed bubble graph depicting the most frequent genomic alterations identified across the 176 patients in the Side-Out cohort (**c**); the dimension of each circle is proportional to the number of occurrences. Red circles indicate genes detected in at least 10% of the study population. Matrix illustrating frequency and co-occurrences of the ten most frequently mutated or amplified genes in the Side-Out cohort (**d** and **e**, respectively).

Between 2019 and 2023, 176 patients were enroled in the Side-Out initiative powered by Perthera. Patients were referred by NCI-designated cancer centres in academic institutions (NCI-CCC) (*n* = 65) as well as community hospitals and large non-NCI-CCC academic centres (*n* = 111). A CONSORT diagram describing the study population is provided in Fig. S2. Genomic profiles were generated for 171 patients enroled in the study, and phospho-proteomic data were collected by two independent laboratories, Neogenomics and Theralink Technologies, for 32 and 69 patients, respectively (Fig. 1a). An overview of the overall study design and approach can be found in Fig. 1b.

The median age of participants at diagnosis was 59 (range 28–85), and the cohort included 173 females and three males (Table 1). Race and ethnicity information were available for 119 patients, of which 96 were White (80.7%), 12 were Black (10.1%), seven were Asians (5.9%) and four were Hispanic (3.4%) (Table 1).

**Table 1:** Demographic and clinical characteristics of patients enroled in the study.

Patients Characteristics (*n* = 176)	
**Age** *(median/range)*	59 (28, 85)

Age by Race/ethnicity *(n, %)*	Frequency
Known	119 (67.6%)
White	96 (80.7%)
Black	12 (10.1%)
Asian	7 (5.9%)
Hispanic	4 (3.4%)
Unknown	57 (32.4%)
**Sex** (*n*, %)	
Female	173 (98%)
Male	3 (1.7%)
**Histology** (*n*, %)	
Known	123 (69.9%)
Ductal	105 (85.4%)
Lobular	10 (8.1%)
Inflammatory	5 (4.1%)
Carcinoma NOS	2 (1.6%)
Ductal/Lobular	1 (0.8%)
Unknown	53 (30.1%)
**Cancer Subtype** (*n*, %)	
HR+/HER2-	110 (62.5%)
TNBC	48 (27.3%)
HR+/HER2+	9 (5.1%)
HR-/HER2+	4 (2.3%)
Unknown	5 (2.8%)
**Stage at diagnosis** (*n*, %)	
Stage 1	11 (6.3%)
Stage 2	22 (12.5%)
Stage 3	17 (9.7%)
Stage 4	115 (65.3%)
Unspecified advanced	6 (3.4%)
Unknown	5 (2.8%)
**Metastatic sites** (*n*, %)	
Known	108 (61.4%)
Bone	15 (13.9%)
Brain	5 (4.6%)
Liver	27 (25.0%)
Lung	4 (3.7%)
Lymph Nodes	21 (19.4%)
Other	13 (12.0%)
More than one site	23 (21.3%)
Unknown	68 (38.6%)
**Biopsy Site** (*n*, %)	
Breast	58 (33.0%)
Liver	36 (20.5%)
Lung	5 (2.8%)
Lymph Node	19 (10.8%)
Brain	6 (3.4%)
Bone	9 (5.1%)
Other	41 (23.3%)
Unknown	2 (1.1%)
**Number of previous treatments** (*n*, %)	
Known	121 (68.7%)
1st line	45 (37.2%)
2nd line	22 (18.2%)
3rd line	13 (10.7%)
4th line	7 (5.8%)
5th line	3 (2.5%)
6th line	4 (3.3%)
Adjuvant	15 (12.4%)
Neoadjuvant	12 (9.9%)
Unknown	55 (31.3%)

Demographic and clinical characteristics of the 176 MBC patients enroled in the study; median and range are reported for patient’s age while counts and frequencies are reported for nominal and ordinal variables.

Of the 176 participants, 110 (62.5%) were affected by HR+/HER2- tumours, 48 (27.3%) by TNBCs, 13 (7.4%) by HER2+ tumours, including nine HR+/HER2+ (5.1%) and four by HER2-enriched (HR-/HER2+) (2.3%) lesions (Table 1).

Of the 176 patients, 65.3% (*n* = 115) had stage 4 metastatic disease at the time of diagnosis. The liver (*n* = 27, 25.0%) was the most frequent site of metastasis, and 23 patients (21.3%) had secondary lesions at more than one site (Table 1). The number of treatments before study participation ranges from one to six (Table 1).

### The *PIK3CA* pathway mutational landscape in a real-world evidence cohort of metastatic breast cancer patients mimics those of well-characterised cohorts

We first assessed the frequencies of the most common genomic alterations in the Side-Out cohort of MBC patients (Fig. 1c; Fig. S3; Fig. S4; Table S1). For nine of 171 patients (5.3%) with genomic data, none of the genes measured by NGS harboured oncogenic alterations (Fig. 1d). As expected, *TP53* (*n* = 75, 43.9%) and *PIK3CA* (*n* = 58, 33.9%) mutations were the most frequently identified pathogenic variants (Fig. 1d), followed by amplifications of *FGF3* (*n* = 24, 14.0%), *FGF4* (*n* = 23, 13.5%), *CCND1* (*n* = 22, 12.9%), *MYC* (*n* = 21, 12.3%) and mutations of *ESR1* (*n* = 21, 12.3%) and *GATA3* (*n* = 19, 11.1%) (Fig. 1d, e). The complete genomic landscape of the entire cohort can be found in Fig. S3 and S4.

**Fig. 2:**
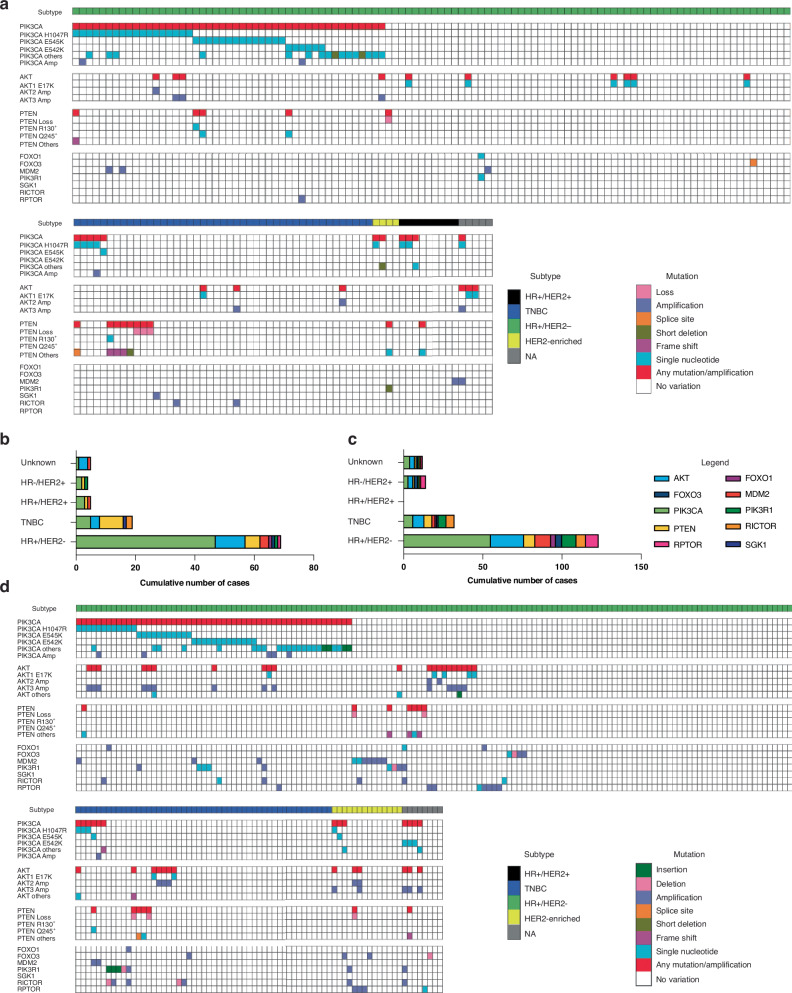
Frequencies of oncogenic alterations of members of the *PIK3CA* pathway in the Side-Out study compared to two cohorts of MBC patients enroled in a single academic institution. Matrix illustrating frequencies of mutations and amplifications across ten members of the *PIK3CA* pathway in the Side-Out cohort (**a**); tumours were subdivided based on the molecular subtypes. HR+/HER2- tumours are shown in the top panel (**a**). Cumulative number of cases with genetic alterations of members of the *PIK3CA* pathway by tumour subtype in the Side-Out cohort (**b**) and the Inserm cohort retrieved from the GENIE database (**c**). Matrix illustrating frequencies of mutations and amplifications across ten members of the *PIK3CA* pathway in the Inserm cohort (**d**); tumours were subdivided based on the molecular subtypes; HR+/HER2- tumours are shown in the top panel.

*PIK3CA* alterations were found in 43.5% of HR+/HER2- tumours (*n* = 47), 50% of HER2-enriched lesions (*n* = 2), 33.3% of HR+/HER2+ (*n* = 3) and 11.1% of TNBCs (*n* = 5) (Fig. 2a). The three most frequent *PIK3CA* gain-of-function pathogenic variants were the *H1047R* (n = 26, 15.2%) mutation of the kinase domain, and the *E545K* (*n* = 15, 8.8%) and *E542K* (*n* = 6, 3.5%) mutations of the helical domain (Fig. 2a). Within the same sample, multiple pathogenic alterations of the *PIK3CA* gene were found in one TNBC (2%) and 10 HR+/HER2- tumours (9%) (Fig. 2a). *PIK3CA* amplifications were found in ~2% (*n* = 3) of patients including two patients with HR+/HER2- MBCs and one patient with a TNBC tumour. *PIK3CA* amplifications were exclusively found in tumours that harboured *PIK3CA* gain-of-function mutations affecting the H1047R and E542K residues. Genetic alterations of the *PIK3CA* gene co-occurred with pathogenic alterations of *ESR1* and *TP53* in 7% (*n* = 12) and 10.5% (*n* = 18) of patients, respectively (Fig. 1d).

Given the primary role of the PI3K/AKT signalling pathway in breast cancer onset and progression, especially in HR+/HER2- tumours, we next extended our analysis to other genes encoding for members of this signalling axis. Of the 92 genes listed in the Pathcards database as members of the *PIK3CA/AKT* ‘SuperPath’ [23], oncogenic alternations of 12 genes, namely *PIK3CA*, *AKT 1*, *2* and *3*, *PTEN, MDM2*, *PIK3R1*, *RICTOR, RPTOR*, *FOXO1, FOXO3* and *SGK1*, were identified in our cohort of patients (Fig. 2a, b).

Oncogenic alterations of genes belonging to the *PIK3CA/AKT* ‘SuperPath’ were identified in 85 (49.7%) of the 171 patients analysed (Fig. 2a). Sixteen of the 171 patients (9.4%) had aberrations of more than one gene belonging to the *PIK3CA* pathway (Fig. 2a). Oncogenic alterations of *AKT1*, *2* and *3* were found in 16 patients (9.4%), including nine *AKT1* E17K point mutations, five *AKT3* and two *AKT2* amplifications (Figs. 1d, 2a). Of interest, *PIK3CA* oncogenic alterations were found in five of the seven patients with amplified *AKT2* or *AKT3*. Loss-of-function alterations of the tumour suppressor *PTEN* were found in 15 patients (8.8%) and co-occurred with *PIK3CA* mutations in five patients. Additional alterations of members of the *PIK3CA/AKT* pathway included mutations of *MDM2* in five patients (2.9%) and of *PIK3R1* and *RICTOR* in two (1.2%) patients. Oncogenic mutations of *FOXO1, FOXO3, RPTOR* and *SGK1* were found in one patient each across the entire cohort (0.6%, respectively) (Fig. 2a).

We next compared mutation rates of members of the *PIK3CA* pathway and their distribution in the Side-Out cohort against two publicly available MBC datasets retrieved from the GENIE database, the Inserm and the MSK cohort (Fig. 1b). While the MSK cohort only included HR+/HER2- tumours (*n* = 1116), biospecimens in the Inserm cohort (*n* = 216) contained all major breast cancer subtypes including 143 (66.2%) HR+/HER2-, 51 (23.6%) TNBC and 14 (6.5%) HER2-enriched MBCs. The molecular subtype of the remaining eight tumours was unknown.

*PIK3CA* pathogenic alterations were found in 31.5% (*n* = 68) of patients in the Inserm cohort and 40.1% (*n* = 448) in the MSK cohort (*p* = 0.03) (Fig. 2c, d, Fig. S5, Table 2). When the analysis was restricted to HR+/HER2- tumours, no differences were detected across the three cohorts (*p* > 0.05; Table 2).

**Table 2:** Pathogenic alterations of the *PIK3CA* pathway in the Side-Out, Inserm and MSK cohorts.

Gene	Side-Out cohort (*n* and %)	Inserm cohort (*n* and %)	MSK cohort (*n* and %)	*p* value
Any mutation^a^	85 (49.7%)	127 (58.8%)	663 (59.4%)	0.06
*AKT*	16 (9.4%)	34 (15.7%)	131 (11.7%)	0.13
*FOXO1*	1 (0.6%)	4 (1.9%)	13 (1.2%)	0.53
*FOXO3*	1 (0.6%)	8 (3.7%)	NA	NA
*MDM2*	5 (2.9%)	12 (5.6%)	71 (6.4%)	0.20
*PIK3CA all subtypes*	58 (33.9%)	68 (31.5%)	448 (40.1%)	*0.03*
*PIK3CA* HR+*/HER2- tumours*	47 (27.5%)	55 (25.5%)	448 (40.1%)	0.71
*PIK3CA amplifications*	3 (1.8%)	6 (2.8%)	14 (1.3%)	0.21
*PIK3R1*	2 (1.2%)	16 (7.4%)	26 (2.3%)	*<0.001*
*PTEN*	15 (8.8%)	14 (6.5%)	95 (8.5%)	0.59
*RICTOR*	2 (1.2%)	13 (6.0%)	25 (2.2%)	*<0.01*
*RPTOR*	1 (0.6%)	12 (5.5%)	25 (2.2%)	*<0.01*
*SGK1*	1 (0.6%)	NA	NA	NA

Counts, frequencies and *p* values comparing oncogenic alterations of different members of the *PIK3CA* pathway in the Side-Out, Inserm and MSK cohorts.

^a^Alterations of any gene included in the PIK3CA/AKT SuperPath.

In the Inserm cohort, *PIK3CA* oncogenic alterations were detected in 55 (38.5%) HR+/HER2- tumours, 6 (11.8%) TNBC, 3 (21.4%) HER2-enriched lesions, four tumours with unknown subtype (Fig. 2c, d). Amplification of the *PIK3CA* gene was observed in 5 HR+/HER2- tumours (3.5%) and one TNBC patient (2%). When the analysis was extended to the other members of the *PIK3CA/AKT* ‘SuperPath,’ members of the *PIK3CA* pathway were found to be altered in 127 patients (58.8%) in the Inserm cohort. Oncogenic alterations of more than one gene were found in 45 biospecimens (Fig. 2d). *AKT* alterations were detected in 34 (15.7%) patients. *PTEN* mutations were present in 14 (6.5%) cases (Fig. 2d). Mutations of *PIK3R1* were found in 16 (7.4%) individuals and of *RICTOR* and *RPTOR* in 13 (6%) and 12 (5.6%) patients, respectively (Fig. 2d). Pathogenic alterations of the transcription factors *FOXO1* and *FOXO3* were found in four (1.9%) and eight (3.7%) patients, respectively.

In the MSK cohort, oncogenic aberrations of the *PIK3CA* pathway were detected in 663 patients (59.4%), of which 148 had alterations of multiple genes. *AKT* was altered in 131 (11.7%) patients and *PTEN* in 95 (8.5%) patients (Fig. S3). Mutations of *PIK3R1* were found in 26 (2.3%) patients and of *RICTOR* (2.2%) and *RPTOR* (2.2%) in 25 patients, respectively. Frequencies of genomic alterations of the main members of the *PIK3CA* pathway were at large similar among the three cohorts, with the exception of a few genes that were relatively infrequent across study sets and of *PIK3CA* when the analysis was not restricted to the HR+/HER2- tumours, which is the only subtype represented in the MSK cohort (Table 2).

### AKT functional activation in metastatic breast cancers cannot be solely predicted by the underlying genomic profile

Next, we assessed whether the underlying genomic profile could predict the activation of critical nodes on the PI3K/AKT/mTOR signalling axis. We used two orthogonal proteomic methods to determine the association between genomic alterations and activation of the PIK3CA/AKT/mTOR pathway with standard IHC and RPPA.

**Fig. 3:**
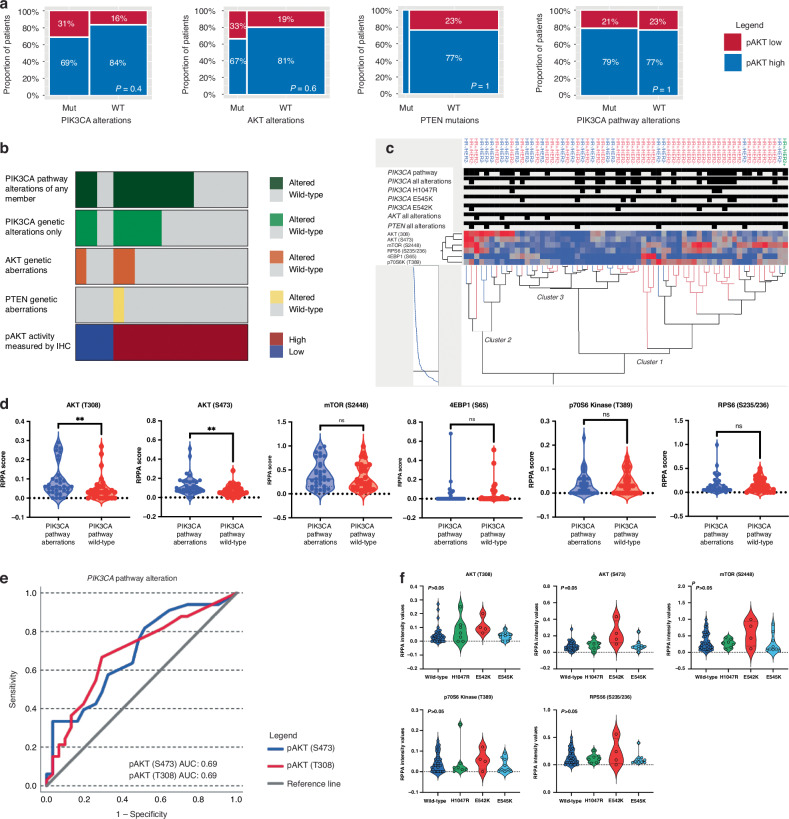
Functional activation of the AKT-mTOR signalling axis in MBCs based on underlying oncogenic alterations of genes encoding for members of the *PIK3CA* pathway. Mosaic plots showing correlations between mutational status and phosphorylated levels of AKT (pAKT) measured by IHC and classified on a dichotomous scale (high versus low) (**a**). The proportion of patients with high AKT activity was first compared between wild-type and *PIK3CA*, *AKT* and *PTEN* mutant/amplified tumours. The analysis was then extended to compare AKT activity in wild-type tumours and lesions with any genetic alterations of the *PIK3CA* pathway (*PIK3CA* pathway alterations). Tile plots summarising frequencies of genetic alterations of the *PIK3CA* pathway along with phosphorylated AKT levels measured by IHC (**b**). Unsupervised hierarchical clustering Ward’s method assessing activation of six members of the PI3K/AKT/mTOR signalling axis in wild-type tumours and lesions harbouring oncogenic alterations of the *PIK3CA* pathway (**c**). Functional protein activation was measured on a continuous scale using RPPA percentile scores. Tumours’ molecular subtypes are listed and colour-coded on the x-axis. On the y-axis, samples with underlying genomic alterations are shown in black. RPPA continuous data are shown on a blue (low activation) to red (high activation) scale. Violin plots comparing activation of the signalling molecules in samples harbouring alterations of any gene of the *PIK3CA* axis and wild-type tumours (**d**); sample median is shown for each plot and asterisks denote comparisons that were statistically different (*p* < 0.01). The receiver operating characteristic (ROC) curve shows the performance of mutations of the *PIK3CA* pathway as potential classifiers for predicting AKT phosphorylation levels in MBCs along with the corresponding area under the curve (AUC) (**e**). Violin plots comparing activation levels of signalling molecules across the three most frequently detected *PIK3CA* oncogenic mutations (**f**).

We first assessed whether AKT (pAKT) activation was more frequently detected in tumours harbouring genetic alterations of the PIK3CA/AKT/mTOR axis compared to wild-type lesions using the IHC data. Of the 32 samples analysed, 25 had high activation of pAKT. The proportion of patients with high pAKT activity was similar in tumours harbouring pathogenic alterations of any member of the *PIK3CA* pathway compared to wild-type tumours (79% versus 77%, respectively; *p* > 0.05) (Fig. 3a). Approximately three quarters (76.9%) of *PIK3CA* wild-type tumours had pAKT levels that mimicked those of mutant lesions suggesting that AKT activity in MBCs is driven by genomic-dependent and independent events (Fig. 3b). Similar associations were also found when the analysis was restricted to the aberration of *PIK3CA*, *AKT* and *PTEN*, individually (Fig. 3a).

We next compared activation levels of six proteins involved in the PI3K/AKT/mTOR signalling axis measured by RPPA on a continuous scale; RPPA values were calculated as percentile scores of the reference population [37]. We first used unsupervised hierarchical clustering to examine how key proteins belonging to the PI3K/AKT/mTOR signalling axis were distributed in 64 samples for which RPPA and NGS data were available (Fig. 3c). At large, subtypes were not associated with AKT/mTOR activation (Fig. 3c, Fig. S6). Samples harbouring mutations of genes encoding for proteins belonging to the PI3K/AKT/mTOR axis were spread across three clusters and had heterogeneous activation of the signalling molecules. Cluster 1 was characterised by increased activation of mTOR and its downstream signalling molecules. Of the 29 patients in this cluster, 15 had genomic alterations of target genes. Cluster 2 was characterised by higher activation levels of AKT and its downstream targets and contained ten samples, including three of the eight tumours harbouring *PTEN* loss of function mutations. Samples included in Cluster 3 had an overall low activation of the different signalling molecules regardless of the mutational status.

**Fig. 4:**
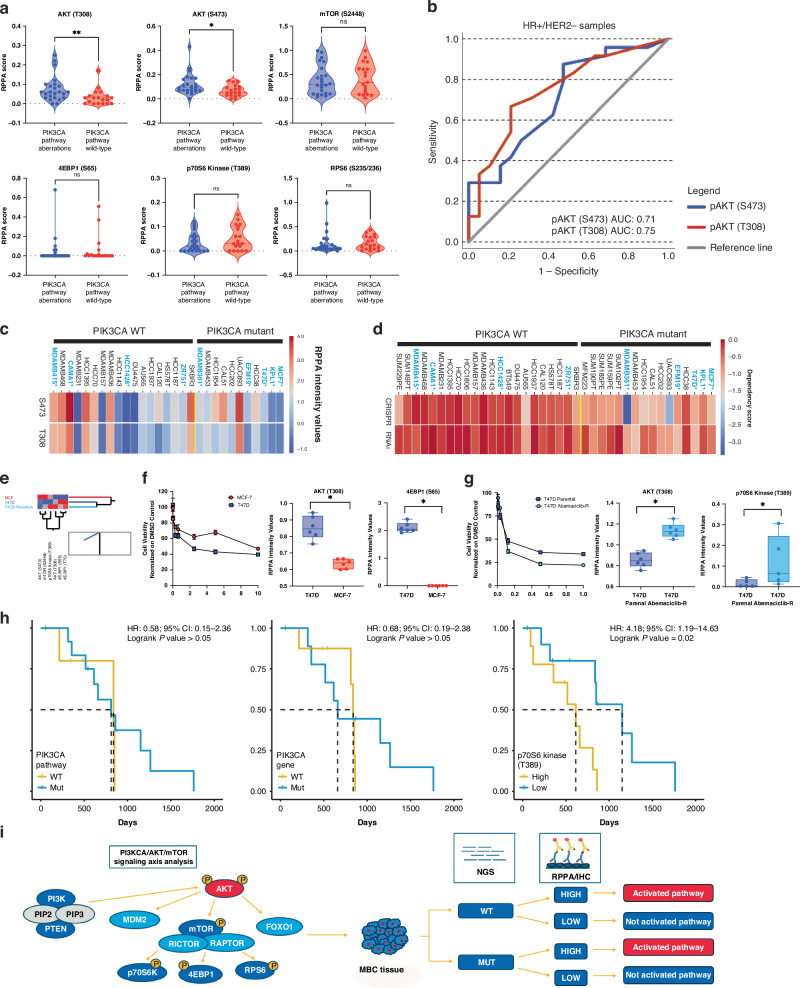
Activation of the AKT-mTOR signalling axis in HR+/HER2- MBCs and their implications for predicting survival to first-line treatment with a CDK4/6 inhibitor in combination with endocrine treatment. Violin plots comparing activation levels, measured as RPPA percentile scores, of signalling molecules in HR+/HER2- MBCs harbouring alterations of any gene of the *PIK3CA* axis (**a**); sample median is shown for each plot, and asterisks denote comparisons that were statistically different (*p* < 0.02). The receiver operating characteristic (ROC) curve shows the performance of mutations of the *PIK3CA* pathway as potential classifiers for predicting AKT phosphorylation levels in HR+/HER2- MBCs along with the corresponding area under the curve (AUC) (**b**). Heat map capturing activation levels of AKT (S473) and (T308) across 29 cell lines publicly available in the DepMap database. HR+/Her2- cells are shown in blue (**c**). Heat map showing *PIK3CA* dependency in 38 cell lines that underwent CRISPR and Ribonucleic acid interference (RNAi) screening (**d**). Unsupervised hierarchical clustering using Ward’s method assessing activation of the PI3K/AKT/mTOR signalling axis in the MCF-7, T47D and T47D abemaciclib-resistant cells; functional protein activation was measured on a continuous scale using RPPA values (**e**). Cell viability line plot of MCF-7 and T47D cells treated with capivasertib (range from 0.01–10 μM) along with box plot depicting the RPPA continuous values of phospho-AKT (T308) and phospho-4EBP1 (S65) and (T70) (*p* < 0.01). Median, highest and lowest values of experimental replicates are shown; asterisks denote comparisons that are statistically significant (**f**). Cell viability line plot of T47D parental and abemaciclib-resistant cells treated with buparlisib (range from 0.007 to 1 μM) along with box plot depicting the RPPA continuous values of phospho-AKT (T308) and phospho-p70S6 Kinase (T389) (*p* < 0.01 and 0.05, respectively). Medians, highest and lowest values of experimental replicates are shown; the asterisks denote comparisons that are statistically significant (**g**). Kaplan-Meier plot along with hazard ratio for progression-free survival in days and 95% confidence interval for patients with genomic alterations of any members of the *PIK3CA* pathway (HR: 0.58; CI: 0.14–2.36), *PIK3CA* (HR:0.67; CI 0.19–2.38), and phospho-p70 S6 Kinase (T389) activity (HR: 4.18; CI: 1.19–14.63) (**h**). P70S6 kinase activity levels were classified on a binary scale (high/low) based on the population median of the continuous RPPA data. Diagram showing a workflow for integrating multi-omic-based profiling for allocating patients to targeted treatments against members of the PI3K/AKT/mTOR pathway (**i**).

When tumours with any mutation of the *PIK3CA* pathway were compared to wild-type lesions, activation of AKT (T308) and (S473) was significantly higher in the mutant population (*p* < 0.01). However, a significant degree of overlap in the samples’ distribution was detected between the two groups (Fig. 3d). ROC analysis assessing the ability of the mutational status to predict AKT activation suggested that pathogenic alterations of the *PIK3CA* pathway are poor predictors of protein activity (AUC: 0.69; Fig. 3e). When the analysis was restricted to *PIK3CA*, *AKT* and *PTEN* individually, activation of the signalling molecules was not different between wild-type and mutant tumours (*p* > 0.05; data not shown). Likewise, when a pathway score was created using the RPPA data as previously described [18], activation of the signalling molecules by RPPA did not differ between the mutant and wild-type populations (data not shown). Similar trends were also observed when the analysis was restricted to the HR+/HER2- population (Fig. 4a, b). Lastly, activation of signalling molecules did not differ between tumours harbouring different *PIK3CA* pathogenic mutations (Fig. 3f) nor in tumours harbouring multiple pathogenic alterations of genes belonging to the *PIK3CA* pathway (Fig. S7).

We next assessed whether *PIK3CA* mutations predicted AKT activation in 29 breast cancer cell lines retreated from the publicly available DepMap portal. At large, activation of AKT was not higher in cell lines harbouring a *PIK3CA* oncogenic mutation (Fig. 4c). We next looked at the significance of *PIK3CA* in relation to the lethality of its deletion or knockdown on a target across all 38 cell lines included in the dataset. Negative dependence scores suggest cell lines are heavily dependent on that gene. In line with our protein data, only two cell lines of the *PIK3CA* mutant cell lines, MDAMB361 and EFM19, showed dependency from the PI3K (Fig. 4d). To confirm the role of signalling molecules in predicting response to agents targeting the PI3K/AKT/mTOR pathway, we next compared the sensitivity to capivasertib in two HR+/HER2- breast cancer cell lines, namely T47D and MCF-7, harbouring hotspot mutations of *PIK3CA* (H1047R and E545K, respectively) [38] with diverse levels of activation of the PI3K/AKT/mTOR axis (Fig. 4e). Compared to the MCF-7, T47D cells had lower IC50 values (2.1 vs. 7.5 μM) and higher levels of phosphorylated AKT (T308) and 4EBP1 (S65) and (T70) (Fig. 4f). Given the increased levels of AKT activity in the T47D cell lines compared to the MCF-7 cells, we next assessed responses to the PI3K inhibitor buparlisib in parental and isogenic abemaciclib-resistant cells. As expected, resistant cells were more sensitive to buparlisib and increased sensitivity was associated with higher levels of phosphorylation of AKT (T308) as well as increased activation of the downstream substrate p70S6 Kinase (Fig. 4g).

Taken together, these data suggest that inferring activation of signalling molecules merely by the underlying genomic alterations of the *PIK3CA* pathway underestimates the activation frequencies of the PI3K/AKT/mTOR axis in MBCs. As AKT and PI3K inhibitors are currently being used in the clinical setting, capturing activity levels of signalling molecules belonging to this axis may help identify MBCs driven by the PI3K/AKT/mTOR signalling pathway and thus may benefit from these treatments.

### Functional activation of AKT downstream substrates is associated with response to first-line treatment in HR+/HER2- metastatic breast cancers

As inhibition of AKT and PI3K activity has recently been shown to delay resistance to ET in combination with a CDK4/6 inhibitor in MBCs in preclinical studies [5, 19], we next assessed whether baseline activation levels of members of the PI3K/AKT/mTOR pathway are associated with response to first-line treatment with a CDK4/6 inhibitor in the Side-Out cohort. Of the 69 patients for which RPPA data were collected, 20 were treated with a CDK4/6 inhibitor in combination with ET in first-line. NGS data were available for 18 of the 20 patients included in this sub-analysis (Table S3). As expected, the mutation status of the *PIK3CA* pathway or *PIK3CA* was not associated with progression-free survival (PFS) (*p* > 0.05) (Fig. 4h). However, patients with p70S6 Kinase (T389) levels above the whole population median had shorter PFS compared to those with low p70S6 Kinase (T389) activity (HR: 4.18, 95% CI:1.19–14.63, *p* = 0.02; Fig. 4h).

Taken together, our data suggest that genomic alterations of the *PIK3CA* pathway are insufficient to predict protein activation in clinical samples and patients’ response to standard of care.

## Discussion

Using a RWE cohort of patients, for which broad molecular profiles were collected in commercial and certified laboratories [20–22], we have conducted a comprehensive analysis to assess the effects of genetic alterations of the *PIK3CA* pathway on PI3K/AKT/mTOR activity in MBCs. Our data suggest that the Side-Out cohort analysed as part of this study has *PIK3CA* genomic pathway profiles similar to those of previously analysed cohorts enroled in elective centres [10, 11, 24–26, 39]. As expected, *PIK3CA* mutations were amongst the most frequently detected pathogenic variants with 33.9% of all tumours and 43.5% of HR+/HER2- MBCs harbouring oncogenic alterations of this key signalling hub [39]. Hotspot mutations on exon 9 (E545K and E542K) and 20 (H1047R) were found in 81% of the mutant lesions, with the H1047R mutation of the kinase domain being the most frequently detected alteration across the 58 mutant specimens (44.8%) [40]. Because most of the genetic alterations detected in our cohort of patients are known to translate into PI3K gain-of-function activity, we next used our sample set to understand the effects these genetic alterations exert on the PI3K/AKT/mTOR signalling axis and the extent to which they can be used to predict pathway activation.

Whether the activation status of AKT was determined using IHC or RPPA-based measurements, genetic alterations of *PIK3CA* and other members of this pathway emerged as poor predictors of protein activity across MBCs. Similarly, a recent analysis assessing AKT activation and its association with *PIK3CA* status in MBCs conducted by Alves et al. has shown that phosphorylation levels of AKT measured by IHC were independent of *PIK3CA* mutational status as high and low AKT levels were found in both mutant and wild-type tumours [19]. Likewise, a secondary biomarker analysis of the FAIRLANE trial, where AKT activity was measured by RPPA and dichotomised based on the population median, showed that 44% of samples with low AKT activity had pathogenic alterations of *PIK3CA/AKT1/PTEN* by NGS or IHC [18]. As the PI3K/AKT/mTOR signalling axis is a central hub where many signal transduction pathways are integrated [41], it is not surprising that the mutational status of the *PIK3CA* axis alone is often insufficient to fully capture the activation status of the PI3K/AKT/mTOR signalling axis.

Over the years, targeting malfunctioning PI3K/AKT/mTOR has been considered an attractive therapeutic target, especially in the MBC metastatic setting, as activation of this signalling axis has been linked to cancer progression and metastasization [10, 42]. Several compounds targeting PI3K have been tested in the clinic, and biomarker analyses have shown that, at large, these compounds are most effective when administered to patients affected by MBC harbouring oncogenic alterations of the *PIK3CA* gene [43]. André and colleagues have shown prolonged PFS and increased clinical benefit in *PIK3CA-*mutated MBC patients treated with alpelisib in combination with fulvestrant compared to patients treated with placebo and fulvestrant [15]. However, when restricted to the *PIK3CA* wild-type cohort of patients, the same proof-of-concept criteria were not met [15]. Similarly, in the BELLE-2 randomised clinical trial assessing response to buparlisib in previously treated HR+/HER2- locally advanced or metastatic breast cancer patients, longer PFS was detected specifically in patients where *PIK3CA* mutations were detected in circulating tumour DNA (ctDNA). These differences were lost in the ctDNA non-mutant group, suggesting that *PIK3CA* status may be a good predictor of response to PI3K inhibitors [44].

However, this association does not appear to be as linear when targeting PI3K downstream signalling molecules [13]. Correlative biomarker analysis of the BOLERO2 trial assessing the efficacy of everolimus in combination with exemestane in HR+/HER2- refractory advanced breast cancers showed that PFS was independent of underlying genetic alteration of *PIK3CA* [13, 39]. Similarly, an exploratory analysis of the CAPItello-291 trial, which led to the approval of capivasertib in combination with fulvestrant for MBC patients whose tumours harbour oncogenic alterations of the *PIK3CA*, *AKT*, or *PTEN* gene, has suggested that capivasertib retains activity beyond AKT-pathway altered tumours [17]. This observation aligns with extensive preclinical work demonstrating that response to capivasertib in breast cancer models is highly dependent upon levels of AKT activity [45, 46] and that phosphorylation of AKT cannot be fully predicted by genomic alterations of its regulators [6, 10, 18].

Aberrant activation of this signalling axis is also a well-known resistance mechanism to various treatments, including ET and CDK4/6 inhibitors [6, 42]. Several studies have shown that inhibition of members of the PI3K/AKT/mTOR can delay the acquisition of resistance to CDK4/6 inhibitors [5, 19]. We have shown that increased activation of the PI3K/AKT/mTOR signalling axis in samples collected from MBC patients that develop disease progression when treated with a CDK4/6 inhibitor in combination with ET extends beyond cancer cells into the surrounding stroma/immune compartment [5]. This suggests that genomic-independent activation of the signalling axis may affect PI3K/AKT/mTOR activity within the tumour microenvironment as a whole [5]. In the current study, we confirm that phosphorylation of signalling molecules, a biochemical event that leads to the activation of members of the PI3K/AKT/mTOR pathway, is associated with response to CDK4/6 inhibitor in combination with ET in MBC patients [9, 47]. However, this association was lost when patients were subclassified based on the mutation status of the *PIK3CA* gene and other members of this signalling axis.

While our study provides strong evidence of the limited extent to which genetic alterations of the *PIK3CA* pathway may predict PI3K/AKT/mTOR activity in MBCs, a few limitations need to be addressed. Enrolment in the Side-Out cohort may be biased toward academically inclined physicians who are more prone to utilise molecular information in their clinical practice. However, given the broad catchment area and number of enroling institutions involved in the study, we believe this cohort is a good representation of heterogeneous populations of MBC patients. In addition, given the RWE nature of the study cohort, capturing standardised and accurate information on patients’ comorbidities and medical history outside of the cancer diagnosis can be challenging. Thus, covariates not accounted for in our study may affect patients’ performance status (and consequently eligibility for some anti-cancer treatment) and outcome, regardless of the underlying biological events driving individual tumours.

Second, tissue samples and molecular profiles were collected at different time points from each patient; thus, correlating molecular data and outcomes associated with specific treatments can be challenging in this cohort. However, this is a common issue related to many biomarker studies, and our cohort is well suited for generating new hypotheses or validating independent findings.

Lastly, as tissues were profiled with different NGS platforms (Fig. S1), the frequency of some of the alterations reported may have been underestimated. However, all platforms captured the most frequently mutated genes and most amplifications. Similarly, for genes belonging to the *PIK3CA* pathway, only genes with low frequencies were not measured by all panels (e.g. *FOXO*, *RICTOR*, *RPTOR*). While these discrepancies may affect our data, the overlap between our cohort and two independent studies suggests that even with these limitations, our work provides an accurate overview of the molecular landscape of MBCs in an RWE.

Despite these limitations, this study reinforces the need for devising multi-omic-based approaches that account for functional signalling data to be incorporated into decision-making pipelines for allocating patients to precision treatments. As the landscape of FDA-approved therapeutics continues to expand for MBC patients, especially for those affected by HR+/HER2− disease, molecularly rationalised treatment selection becomes gradually more important to effectively allocate patients to treatment. Given the recent approval of agents targeting the PI3K/AKT/mTOR axis, our data provide timely and important insights on the role of multi-omic profiling for MBC patients, even if based on an interim analysis of a growing RWE cohort. With different assays available in commercial laboratories designed to capture expression and activation levels of these drug targets and their substrates, functional data should routinely be evaluated as an integral part of a tumour’s molecular landscape to guide treatment selection for MBC patients (Fig. 4i) [20, 48, 49].

## Supplementary information

Supplementary material

## Data Availability

Molecular profiles for patients in the Side-Out cohort can be accessed at https://sideoutfoundation.gmu.edu/. Data from the GENIE cohort were accessed at https://www.cbioportal.org/. CRISPR- and RNAi-based *PIK3CA* dependency scores and RPPA-based measurements of phosphorylated AKT (S473 and T308) for 38 cell lines were obtained from Cancer Dependency Map (DepMap) Data Explorer (https://depmap.org/).
