# Supplementary material for: Functional activation of the AKT-mTOR signalling axis in a real-world metastatic breast cancer cohort
Source: Br J Cancer. 2024 Sep 25;131(9):1543–54. doi: 10.1038/s41416-024-02852-y (PMC11519601; doi:10.1038/s41416-024-02852-y)

Supplementary Table 1. Frequencies of oncogenic alterations, including mutations and structural variants, in the 171 patients enrolled in the study for which NGS data were available.

Gene	Number of patients with aberration in SOF cohort	Frequency of aberration (%)
TP53	77	45.0
PIK3CA	58	33.9
ESR1	25	14.6
FGF3	24	14.0
FGF4	23	13.5
CCND1	22	12.9
GATA3	22	12.9
MYC	21	12.3
FGFR1	18	10.5
FGF19	17	9.9
ARID1A	16	9.4
HER2	15	8.8
PTEN	15	8.8
ZNF703	15	8.8
RB1	14	8.2
MCL1	12	7.0
RAD21	12	7.0
BRCA1	10	5.8
CDH1	10	5.8
AKT1	9	5.3
MAP3K1	9	5.3
No Significant Variants	9	5.3
BRCA2	8	4.7
EMSY	8	4.7
KAT6A	8	4.7
KMT2C	8	4.7
NF1	8	4.7
MAP2K4	7	4.1
ZNF217	7	4.1
DNMT3A	6	3.5
EP300	6	3.5
FGFR2	6	3.5
NSD3	6	3.5
AKT3	5	2.9
ARFRP1	5	2.9
AURKA	5	2.9
CHEK2	5	2.9
EGFR	5	2.9
KRAS	5	2.9
MDM2	5	2.9
NTRK1	5	2.9
ASXL1	4	2.3

ATM	4	2.3
BCORL1	4	2.3
BTG2	4	2.3
CCNE1	4	2.3
CDKN2A	4	2.3
CREBBP	4	2.3
GNAS	4	2.3
KMT2D	4	2.3
NOTCH2	4	2.3
PALB2	4	2.3
SF3B1	4	2.3
SMAD4	4	2.3
SPEN	4	2.3
CDK12	3	1.8
CDKN1B	3	1.8
CTNNA1	3	1.8
FGFR4	3	1.8
KDM6A	3	1.8
MSH2	3	1.8
MUTYH	3	1.8
NBN	3	1.8
NOTCH1	3	1.8
PIK3C2B	3	1.8
SOX2	3	1.8
SPOP	3	1.8
TBX3	3	1.8
TNFAIP3	3	1.8
TOP2A	3	1.8
AKT2	2	1.2
ATR	2	1.2
BCL2L2	2	1.2
BRIP1	2	1.2
CHEK1	2	1.2
CKS1B	2	1.2
CTCF	2	1.2
EPHA3	2	1.2
ERBB3	2	1.2
EZH2	2	1.2
FANCC	2	1.2
FBXW7	2	1.2
JAK2	2	1.2
KDM5C	2	1.2
KIT	2	1.2
LYN	2	1.2
MDM4	2	1.2
MSH3	2	1.2
MSH6	2	1.2

MYB	2	1.2
MYCL	2	1.2
NFKBIA	2	1.2
NOTCH3	2	1.2
PARP1	2	1.2
PBRM1	2	1.2
PIK3R1	2	1.2
PRKDC	2	1.2
PTCH1	2	1.2
RAF1	2	1.2
RASA1	2	1.2
RICTOR	2	1.2
RUNX1	2	1.2
SETD2	2	1.2
SMARCA4	2	1.2
STK11	2	1.2
TET2	2	1.2
ACVR1B	1	0.6
AMER1	1	0.6
APC	1	0.6
ATRX	1	0.6
BAP1	1	0.6
BARD1	1	0.6
BCL2L1	1	0.6
BLM	1	0.6
CARD11	1	0.6
CASP8	1	0.6
CBFB	1	0.6
CCND2	1	0.6
CCT6B	1	0.6
CD274	1	0.6
CD79B	1	0.6
CDK4	1	0.6
CDK6	1	0.6
CEBPA	1	0.6
CHD2	1	0.6
CTNNB1	1	0.6
CYLD	1	0.6
DDR2	1	0.6
EBF1	1	0.6
EED	1	0.6
ERCC4	1	0.6
ERRFI1	1	0.6
FANCD2	1	0.6
FAT1	1	0.6
FBXO11	1	0.6
FGF10	1	0.6

FGF14	1	0.6
FGF23	1	0.6
FGF6	1	0.6
FH	1	0.6
FLCN	1	0.6
FLT4	1	0.6
FOXO1	1	0.6
FOXO3	1	0.6
FRS2	1	0.6
GATA6	1	0.6
GID4	1	0.6
GNA13	1	0.6
GRIN2A	1	0.6
H3F3A	1	0.6
HDAC1	1	0.6
HDAC4	1	0.6
HGF	1	0.6
HNF1A	1	0.6
IGF1R	1	0.6
IKBKE	1	0.6
IRF4	1	0.6
IRS2	1	0.6
JAK1	1	0.6
KDM5A	1	0.6
KEAP1	1	0.6
KEL	1	0.6
KMT2A	1	0.6
LRP1B	1	0.6
LRRK2	1	0.6
MAPK3	1	0.6
MITF	1	0.6
MLH1	1	0.6
MLH3	1	0.6
MLL3	1	0.6
MRE11	1	0.6
NCOR2	1	0.6
NF2	1	0.6
PARK2	1	0.6
PARP2	1	0.6
PD-L1	1	0.6
PD-L2	1	0.6
PKN1	1	0.6
PLCG2	1	0.6
POLD1	1	0.6
PRDM1	1	0.6
PRKAR1A	1	0.6
PRKCI	1	0.6

PRKN	1	0.6
RAD51B	1	0.6
RAD51C	1	0.6
RECQL4	1	0.6
RNF43	1	0.6
ROS1	1	0.6
RPTOR	1	0.6
SDHC	1	0.6
SGK1	1	0.6
SMAD3	1	0.6
SMC1A	1	0.6
SOCS1	1	0.6
SUFU	1	0.6
TERC	1	0.6
TNFRSF14	1	0.6
TP63	1	0.6
TTF1	1	0.6
TYRO3	1	0.6
WHSCL1L1	1	0.6
XRCC2	1	0.6
YEATS4	1	0.6
YES1	1	0.6
ZNRF3	1	0.6

Supplementary Table 2. Description of clinical-pathological characteristics, treatment history, and key oncogenic alterations in tissue samples collected from 20 MBC patients who received first-line treatment with a CDK4/6 inhibitor in combination with endocrine therapy.

Patient characteristics (N=20)	Frequency
**Subtype**	
HR+/HER2-*	19 (95%)
HR+/HER2+	1 (5%)
**Combination of treatment**	
Abemaciclib + ET NOS	1
Palbociclib	2
Palbociclib/Abemaciclib + Letrozole	1
Palbociclib + Anastrozole	1
Palbociclib + ET NOS	1
Palbociclib + Exemestane	1
Palbociclib + Fulvestrant	5
Palbociclib + Fulvestrant + ZN-C5	1
Palbociclib + Letrozole	5
Palbociclib + Letrozole + Fulvestrant	1
Ribociclib + Letrozole + Alpelisib	1
**CDK4/6 inhibition associated alterations**	
AKT 1	1 (5.6%)
CCND1	2 (11.1 %)
ESR1	6 (33.3%)
PIK3CA	10 (55.6%)
RB1	1 (5.6%)

* The tumors of three patients, originally classified as a HR+/HER2- tumor lost the ER receptor at later time and were classified as TNBCs.

Supplementary Table 3. Treatment history for patients treated with a CDK4/6 inhibitor in combination with ET stratified by p70S6K (T389). In bold are highlighted treatments targeting the PI3K/AKT/mTOR signaling axis.

Patient ID	p70S6K (T389) status	Line of Treatment	Treatment Received
04-00090	Above Median	1st line	Palbociclib + Letrozole
		2nd line	**Capivasertib (vs placebo) + Fulvestrant**
04-00127	Above Median	1st line	Palbociclib + Anastrozole
		2nd line	**Alpelisib + Fulvestrant**
		Radiation 1	RTx
04-00128	Above Median	1st line	Palbociclib + Letrozole
		2nd line	**Alpelisib**
		3rd line	Capecitabine
		Adjuvant1	Anastrozole
		Neoadjuvant 1	Anastrozole + Fulvestrant
04-00138	Above Median	1st line	Palbociclib + Fulvestrant
		2nd line	**Everolimus + Exemestane**
		3rd line	Capecitabine
		4th line	Docetaxel
		5th line	Doxorubicin
		6th line	Gemcitabine
		Adjuvant 1	Doxorubicin + Cyclophosphamide
		Adjuvant 2	Tamoxifen + Anastrozole + Letrozole
04-00146	Above Median	1st line	**Ribociclib + Letrozole + Alpelisib**
		2nd line	Paclitaxel
		3rd line	Fulvestrant
		4th line	Vinorelbine + Trastuzumab + Pertuzumab
		5th line	Peg-Doxorubicin
04-00154	Above Median	1st line	Palbociclib + Exemestane
		2nd line	Capecitabine
		3rd line	Gemcitabine + Paclitaxel
		Adjuvant 1	Docetaxel + Cyclophosphamide
		Adjuvant 2	Gemcitabine + Vincristine + Doxorubicin
04-00155	Above Median	1st line	Palbociclib + Fulvestrant
		2nd line	Capecitabine + RT
		Neoadjuvant 1	Doxorubicin + Cyclophosphamide + Paclitaxel
04-00225	Above Median	1st line	Palbociclib + Fulvestrant
		2nd line	Paclitaxel
		3rd line	**Alpelisib**
		Radiation 1	RTx
04-10073	Above Median	1st line	Exemestane
		1st line	Palbociclib + Fulvestrant
04-00094	Below Median	1st line	Palbociclib + Letrozole

04-00119	Below Median	1st line	Palbociclib + Letrozole
		2nd line	**Alpelisib + Fulvestrant**
04-00133	Below Median	1st line	Palbociclib + Fulvestrant + ZN-C5
		2nd line	**Everolimus + Exemestane**
		3rd line	Capecitabine
		Adjuvant 1	Letrozole + Anastrozole
		Adjuvant 2	Cyclophosphamide + Methotrexate + 5FU
04-00136	Below Median	1st line	Palbociclib + Letrozole + Fulvestrant
		2nd line	Capecitabine + Fulvestrant
		3rd line	Abemaciclib + Fulvestrant
		5th line	**Everolimus + Fulvestrant**
		4th line	**Alpelisib + Fulvestrant**
		6th line	Carboplatin + Gemcitabine
04-00226	Below Median	1st line	Palbociclib + Abemaciclib + Letrozole
		2nd line	Gemcitabine + Carboplatin + Pembrolizumab
04-00247	Below Median	1st line	Palbociclib
		Neoadjuvant 1	Doxorubicin + Cyclophosphamide + Paclitaxel
		Radiation 1	RTx
		Radiation 2	RTx
04-00258	Below Median	1st line	Palbociclib
		Neoadjuvant 1	Doxorubicin + Cyclophosphamide
		Radiation 1	RTx
		Radiation 2	RTx
04-10070	Below Median	1st line	Abemaciclib + ET
		2nd line	**MLN0128 + Alpelisib + ET**
		3rd line	Atezolizumab + Nab-Paclitaxel + Olaparib
		4th line	Abemaciclib + ET
04-10074	Below Median	1st line	Palbociclib + Letrozole
		2nd line	Capecitabine
04-10077	Below Median	1st line	Palbociclib + ET
		Adjuvant 1	Cyclophosphamide + Docetaxel + Tamoxifen
04-10090	Below Median	1st line	Palbociclib + Fulvestrant
		2nd line	Methotrexate + Cyclophosphamide
		3rd line	Palbociclib + Rintodestrant + Estradiol
		4th line	Rebastinib + Paclitaxel
		Adjuvant 1	Capecitabine + RT
		Neoadjuvant 1	Doxorubicin + Cyclophosphamide + Paclitaxel + RT

Supplementary Figure 1. Venn diagram illustrating frequencies of overlapping genes across NGS platforms for the entire gene panels (Panel A) and members of the *PIK3CA* pathway (Panel B). FM: Foundation Medicine; PGDX: Personal Genome Diagnostics.

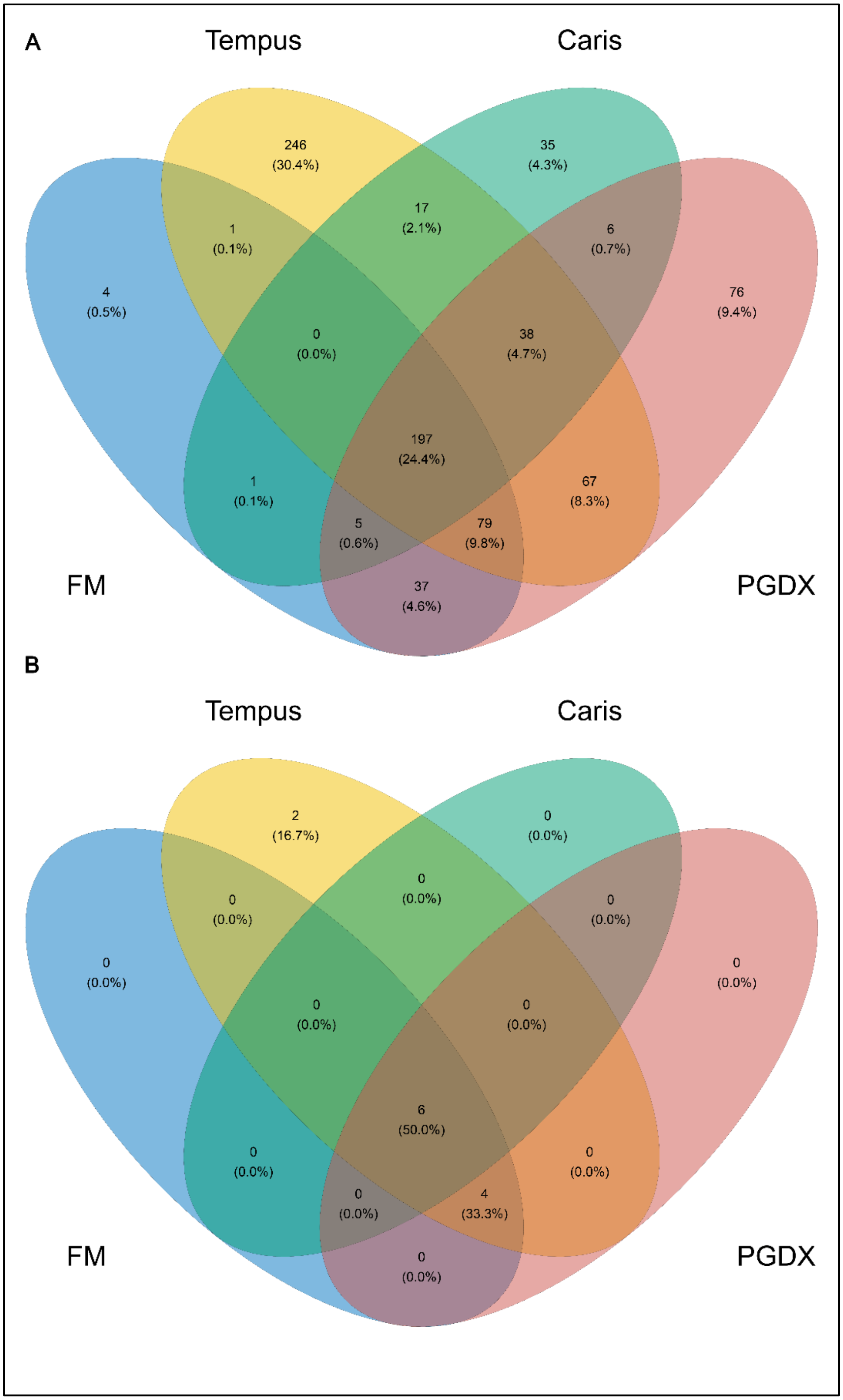

Supplementary Figure 2. CONSORT diagram capturing the demographic and molecular characteristics of the RWE cohort.

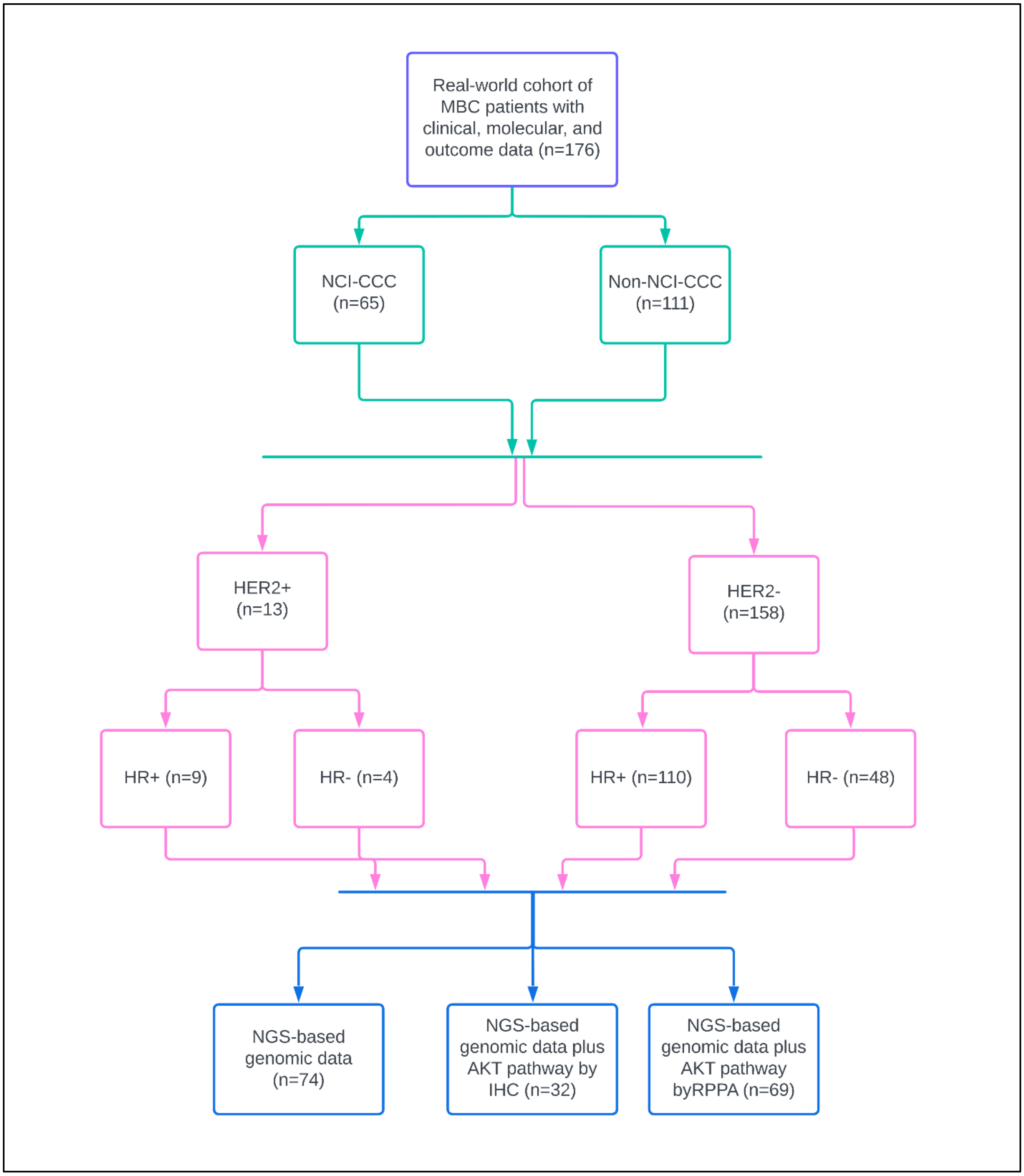

Supplementary Figure 3. Matrix capturing short variant mutations of patients enrolled in the Side-Out cohort based on molecular subtype.

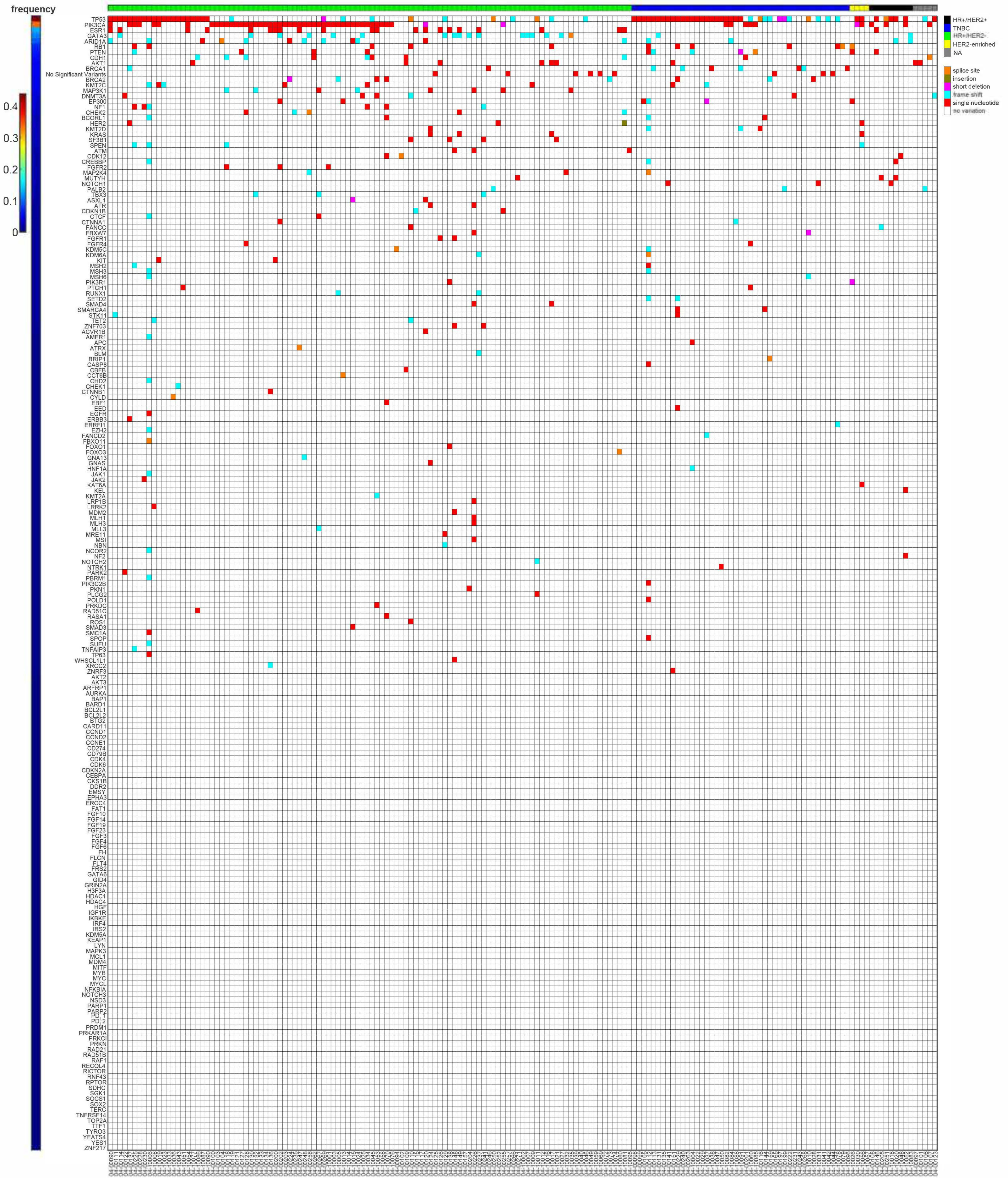

Supplementary Figure 4. Matrix illustrating structural genetic alterations for each patient enrolled in the Side-Out cohort based on their molecular subtype.

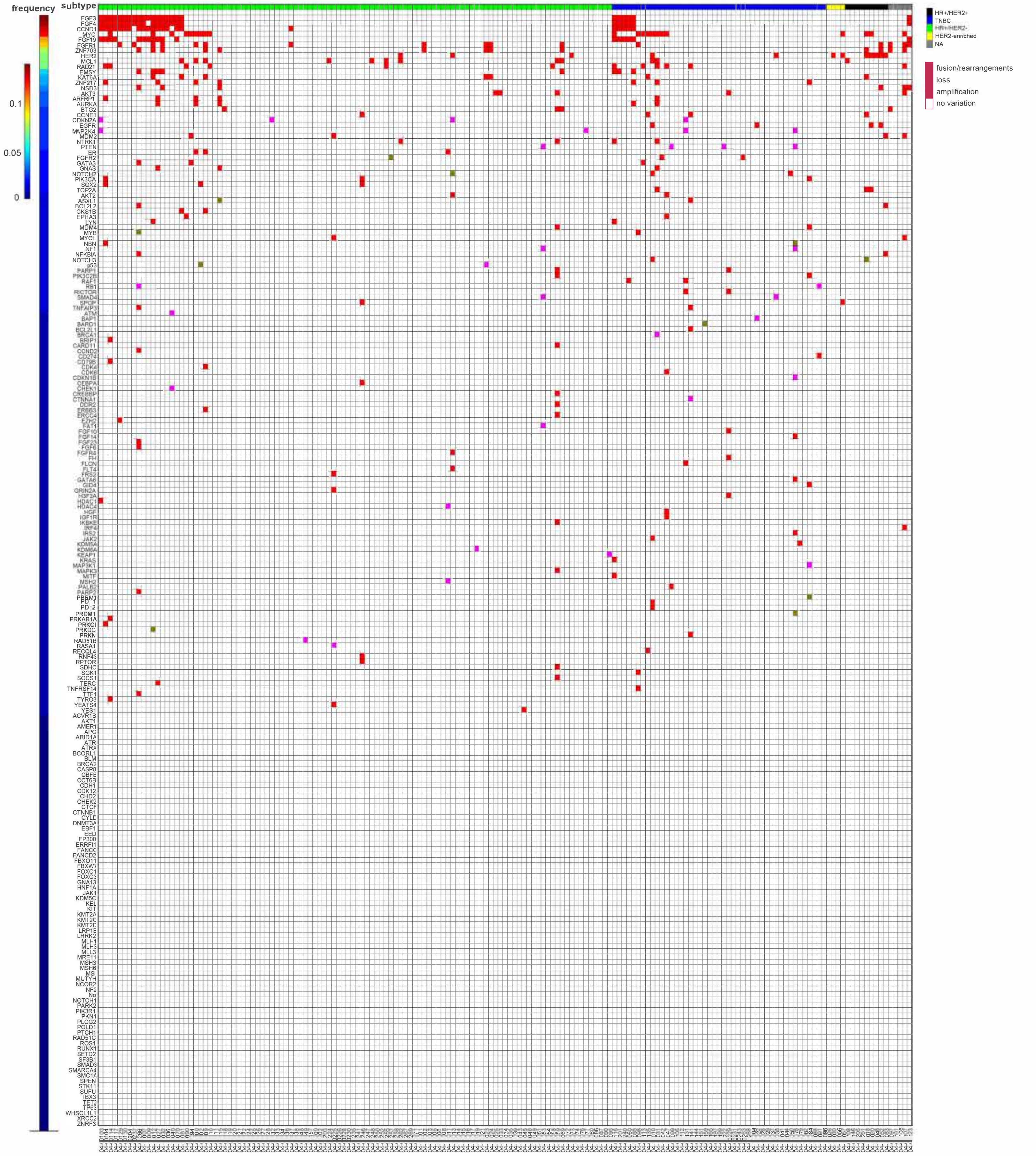

Supplementary Figure 5. Matrix illustrating genomic alterations of different members of the *PIK3CA* pathway in the HR+/HER2- MSK cohort.

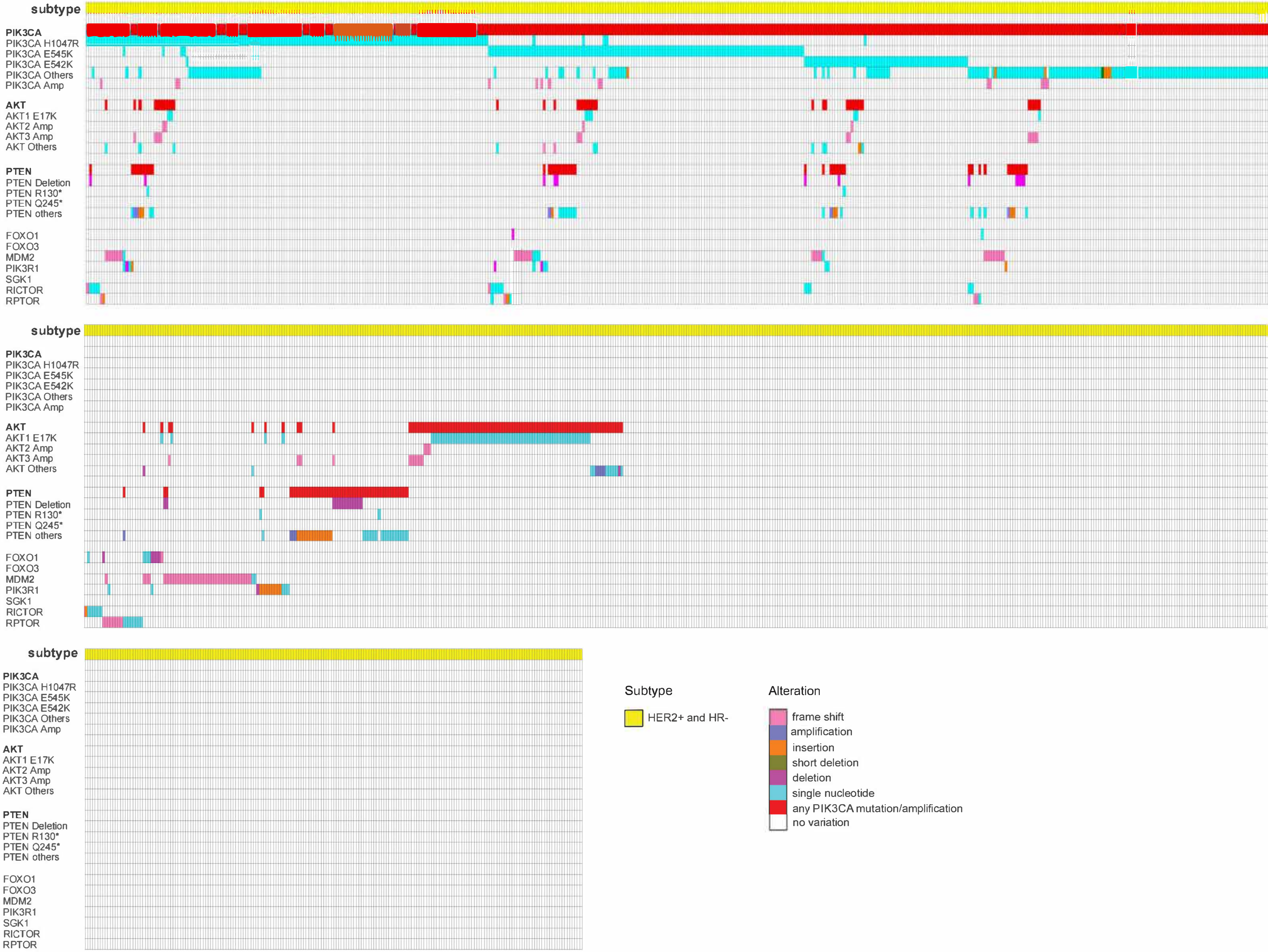

Supplementary Figure 6. Activation levels of six signaling molecules belonging to the PI3K/AKT/mTOR axis measured by RPPA in HR+/HER2- and triple negative MBCs.

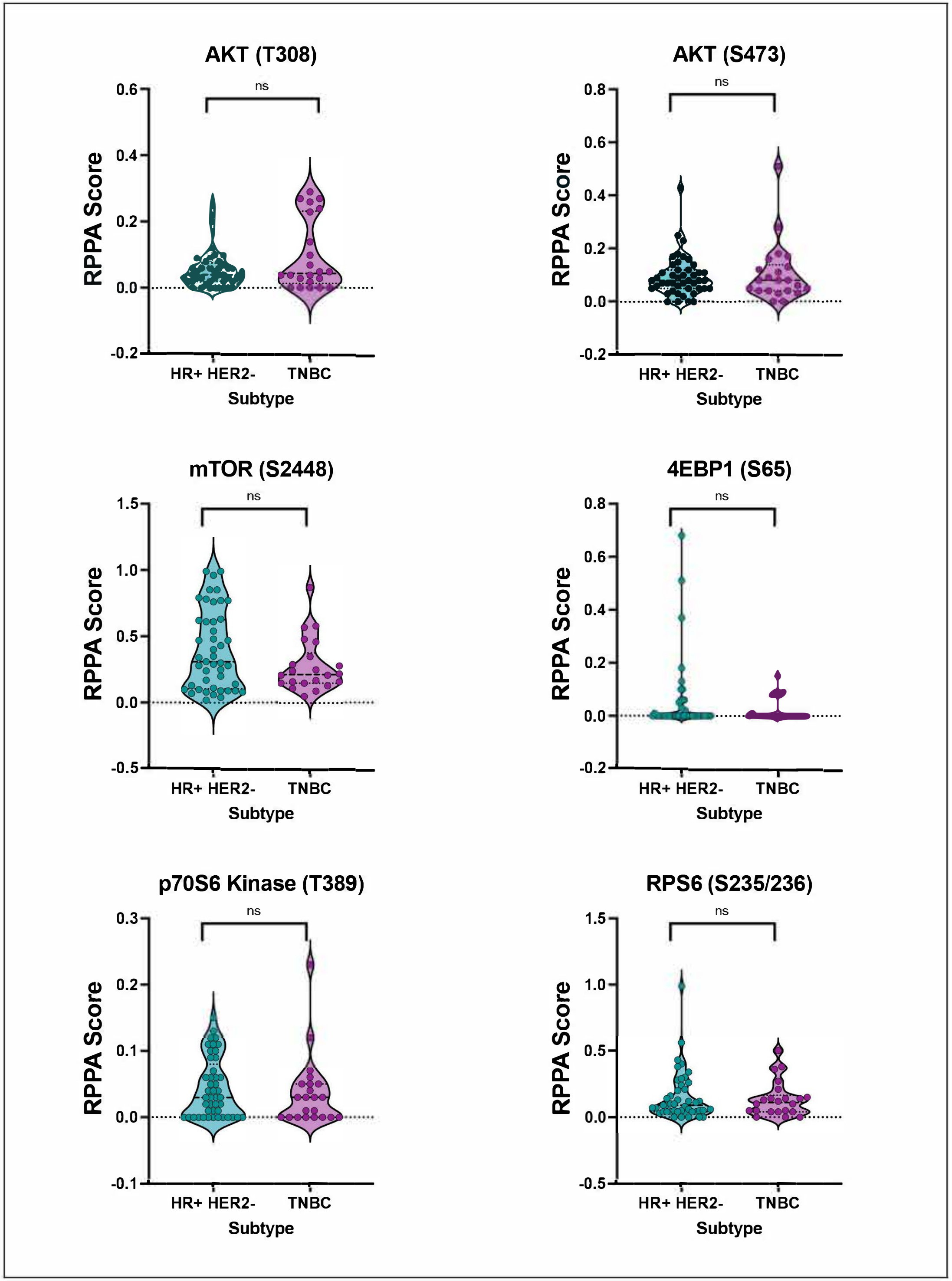

Supplementary Figure 7. Activation levels of six signaling molecules belonging to the PI3K/AKT/mTOR axis across wild-type MBCs and tumors with one or more alterations of members of the *PIK3CA* axis. Yellow circles denote *PIK3CA* amplifications.

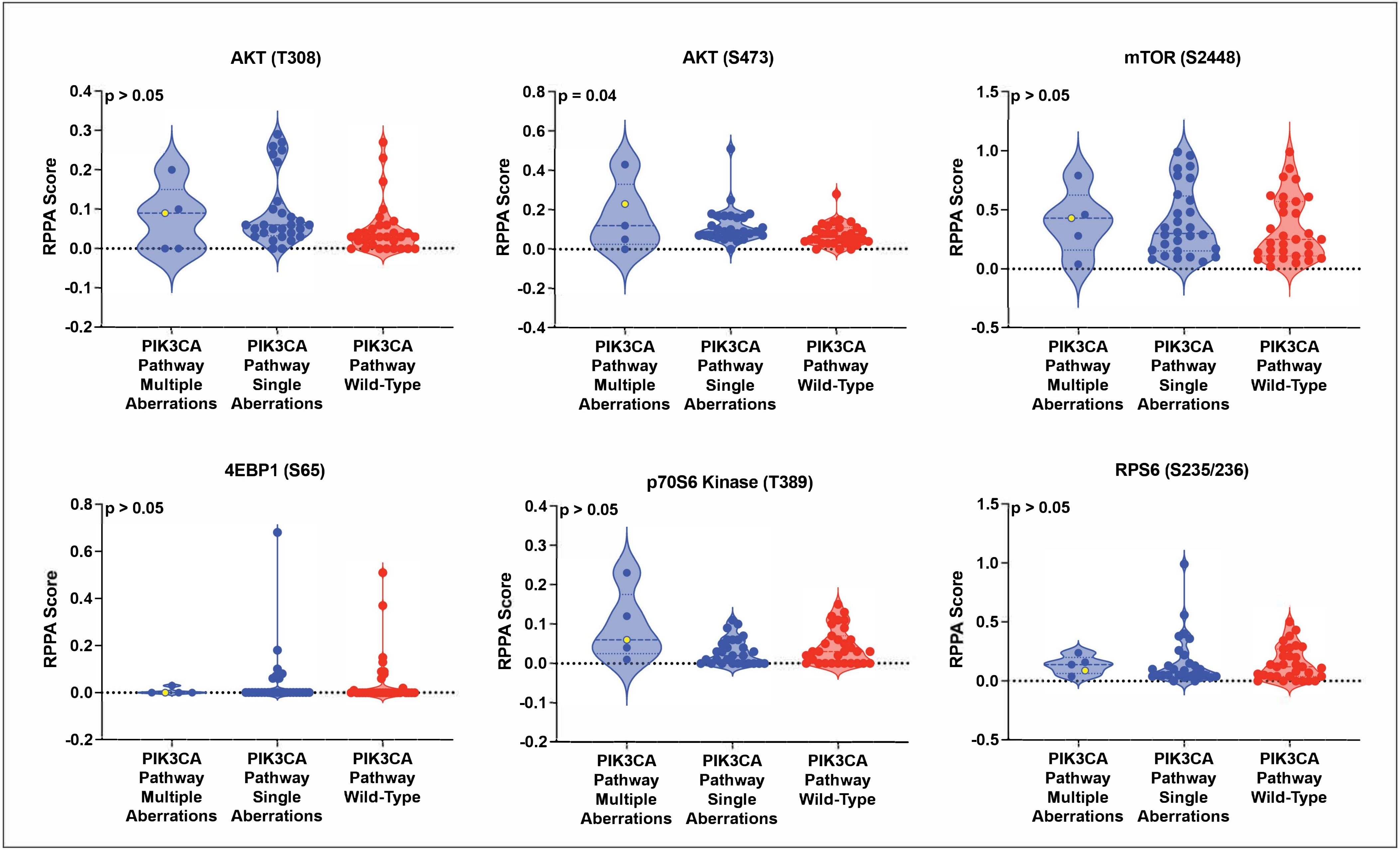

Supplement: Supplementary file 1 — Supplementary material [file 41416_2024_2852_MOESM1_ESM.pdf]
